# Physical Growth and Body Adiposity Curves in Students of the Maule Region (Chile)

**DOI:** 10.3389/fped.2019.00323

**Published:** 2019-08-06

**Authors:** Rossana Gomez-Campos, Miguel Arruda, Cynthia Lee Andruske, Daniel Leite-Portella, Jaime Pacheco-Carrillo, Camilo Urral-Albornoz, Jose Sulla-Torres, Cristian Luarte-Rocha, Marco Antonio Cossio-Bolaños

**Affiliations:** ^1^Departamento de Diversidad e Inclusividad Educativa, Universidad Católica del Maule, Talca, Chile; ^2^Faculty of Physical Education, State University of Campinas, São Paulo, Brazil; ^3^Centro de Investigación CINEMAROS, Arequipa, Peru; ^4^Faculdade de Educação Física, Universidade Municipal de São Caetano do Sul, São Paulo, Brazil; ^5^Departamento de Ciencias de la Educación, Universidad de Bio Bio, Chillán, Chile; ^6^Escuela de Kinesiología, Facultad de Salud, Universidad Santo Tomás, Talca, Chile; ^7^Universidad Nacional de San Agustín de Arequipa, Arequipa, Peru; ^8^Facultad de Ciencias de la Educación, Universidad San Sebastián, Concepción, Chile; ^9^Programa de Doctorado en Ciencias de la Actividad Física, Universidad Católica del Maule, Talca, Chile

**Keywords:** physical growth, adiposity, curves, children, adolescents

## Abstract

**Objectives:** Physical growth and body adiposity patterns provide relevant information to infer the nutritional and health status of students. Our objectives were (a) to compare the variables of body adiposity and physical growth of Chilean children and adolescents with data from the CDC-2012 and international studies, and (b) to develop regional reference curves to evaluate growth and body adiposity.

**Methods:** 8,261 children and adolescents were studied. We evaluated the weight, height, and waist circumference (WC). The Body Mass Index (BMI) was calculated. Their physical growth and body adiposity were compared with the CDC-2012 references as well as with other international references. Percentile curves for weight, height, BMI, and WC were constructed with the LMS method.

**Results:** The Chilean students showed reduced weight and height during adolescence when compared with the CDC-2012 reference. During early ages, the BMI for the Chilean sample was lower while at advanced ages, the WC values were greater in comparison to the CDC-2012 reference. Graphic comparisons with international studies indicated that Chilean students weighed more at all ages. However, height was slightly greater until age 14 for males and age 11 for females. Body adiposity (BMI and WC) for the Chilean students was slightly higher at early ages while at later ages, adiposity values were relatively similar for both sexes.

**Conclusions:** Discrepancies were observed between the physical growth and body adiposity trajectories and the American CDC-2012 references and the international studies. The proposed percentiles for weight, height, BMI, and WC for each age and sex may be useful for health sciences professionals and researchers.

## Introduction

Physical growth patterns can be used as indicators of health and well-being, reflecting nutrition, and living conditions (1). Height and weight are considered the most important indicators of physical growth (2).

Graphs that present reference values are an important tool used to assess and monitor the individual's growth (3) and that of the population in general (4). Moreover, measuring body adiposity is critical to assess the degree to which different populations are rated with regard to excess weight (5).

The importance of said evaluation emerges stemming from the unfortunate increase in overweight and obesity in children and adolescents (6). The most common methods for evaluation include: BMI, Waist Circumference (WC), Waist-Hip Ratio, Waist-to-Height Ratio, and skinfold thickness, among others.

Generally, several international benchmarks exist that allow the observation of physical growth through weight and stature (7, 8) as well as body adiposity through BMI and WC (5, 9, 10). Recently, the updated standards that allow tracking and sorting of physical growth, nutritional status, and body adiposity in children, adolescents, and adults from the United States of America were published by the Center for Disease Control and Prevention (CDC-2012) (11). This reference provides variables such as weight, height, skinfolds, circumferences, and lengths of body segments (12).

In fact, thus far, the Chilean Ministry of Health, through the Technical Prescript for Nutritional Evaluation in force for children and adolescents ages 6–18 years old (13), suggests the use of the CDC-2000 (7) references to classify physical growth. For the purposes of classification of abdominal adiposity, it is recommended to use the American curves, as described by Fernández et al. (5), to evaluate and interpret WC. However, the CDC-2012 curve (11) is currently not considered for evaluating growth, nutritional status, and body adiposity, despite being updated in relation to other international references.

Therefore, before the need to use only one reference to assess weight, height, BMI, and WC of children and adolescents in the Maule Region (Chile), the purpose of this study was to collect the physical growth and body adiposity variables in order to determine the magnitude of the distribution of physical growth and overweight in students. To this end, we proposed two objectives: (a) to compare the physical growth and body adiposity in children and adolescents of the Maule Region (Chile) with the American CDC-2012 reference and other international studies, in such a way that this information might be relevant and reflect the differences between populations and consequently the need (b) to develop regional curves to classify the physical growth and body fat in children and adolescents.

## Materials and Methods

The research carried out for this study was descriptive and cross-sectional. Before starting the data collection, all the parents were informed in detail about the goals of the research, and then they signed consent forms and authorized the participation of their children.

This study received approval from the respective school authorities as well as the Ethics Committee from the Universidad Autónoma of Chile (protocol no. 238/2013), and the research was conducted according to the principles of the Declaration of Helsinki.

### Subjects/Sample

The sample population was composed of 31,696 students ranging in age from 6.0 to 18.9 years. The students belonged to 12 municipal elementary and high schools from four provinces in the Maule Region, Chile (Cauquenes, Curicó, Linares, and Talca).

Probabilistic sampling (random) was used to calculate the sample size, resulting in a sample of 9,232 subjects [4,851 (15.3%) males and 4,381 (13.8%) females] at 95%CI. The mean age was 13.73 (± 2.91) for males and 13.51 (± 3.10) for females.

**Figure 1:**
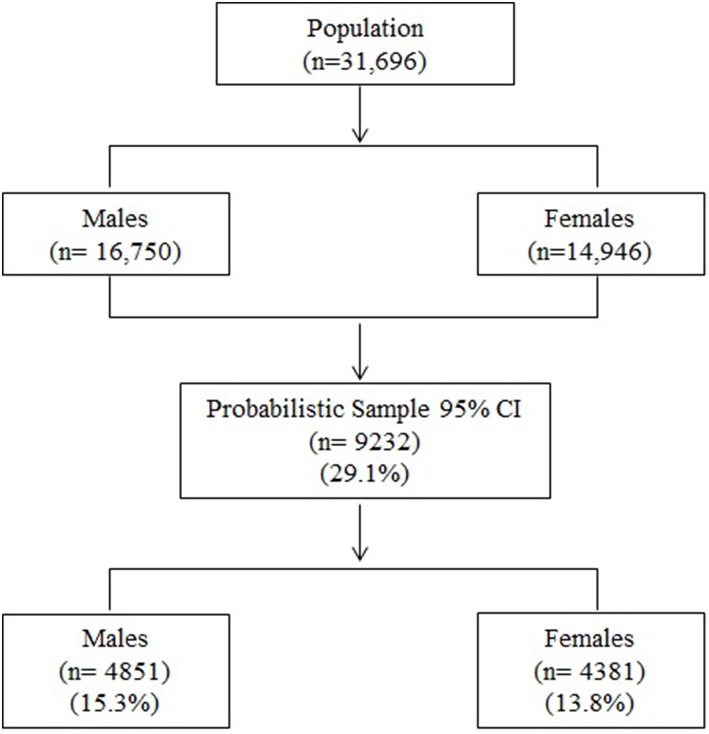
Organization process for selection of the sample.

Figure 1 shows the procedure for the sample selection.

### Measures

All data collection procedures were performed in the laboratory of the Universidad Autónoma de Chile (Talca, Chile). Anthropometric evaluations were conducted from 8:30 a.m.−12:30 p.m. and from 2:30–6:00 p.m., Monday to Friday, from March to November of 2014 and 2015. The evaluations were conducted by experienced professionals trained in the necessary evaluation procedures (six professionals).

The date of birth information for students (day, month, and year) was provided by the administration of the schools. Students were grouped in 13 categories by age group in 1 year intervals (ex.: age 6.0–6.9; age 7.0–7.9…).

To measure the anthropometric variables, the standardized protocol by Ross and Marfell-Jones (14) was used. Body weight (Kg) was assessed using an electronic scale (Tanita, United Kingdom, Ltd.) with a range of 0–150 Kg and precision of 100 g. Standing height was measured with a portable stadiometer (Seca Gmbh & Co. KG, Hamburg, Germany) with a precision of 0.1 mm, according to the Frankfurt plane. WC (cm) was measured at the midpoint between the lower ribs and the top of the iliac crest with a metal anthropometric measuring tape, Seca brand, graduated in millimeters with a precision of 0.1 cm. The Body Mass Index (BMI) was calculated using the formula: BMI = weight (kg)/stature^2^ (m).

To ensure quality control of the anthropometric measurements, two anthropometric measurements were conducted every 10 subjects (*n* = 862 subjects). This information allowed the identification of the technical error inter- and intra-evaluator. It showed values inferior to 3%.

Comparisons of physical growth and body adiposity were made with international references: CDC-2012 (11), Alfaro et al. (15) from Argentina, Chaves et al. (3) from Portugal, Gomez-Campos et al. (16) from Brazil, Fernández et al. (5) from the USA, and Vargas et al. (17) from Venezuela.

### Statistical s Analysis

The normal distribution of the data was verified using the Kolmogorov-Smirnov test. The descriptive statistics were calculated in mean, standard deviation (SD), and ranges. Differences between males and females were verified through the *t*-test for independent samples. Z-scores were calculated with the goal of comparing data from physical growth and body adiposity of the students from the present study with the normative references established by the CDC 2012. The following equation was used to calculate the Z-scores: Z = [(X /M) L – 1] / L ^*^ S, where X was the measure observed (weight, height, BMI, and WC) for each subject; M the median, L the asymmetry value, −1 the constant, and S the coefficient of variation. The L, M, and S values were obtained from the normative tables from the CDC-2012. When the L, M, and S values were not available, the Z-scare was used: Z = (X – M) / SD, where X is the observed value, M the average, and SD the standard deviation. M and SD were obtained from the CDC-2012 reference for age and sex.

The significance value adopted was *p* < 0.05. Comparisons with other international references were graphically represented using the 50th percentile. Smoothed percentile curves were created for weight, height, BMI, and WC by age group and sex, based on the LMS method (9). The software LMS Chart Maker version 2.3 (18) was used. The final percentile curves were smoothed to create three specific curves by age: L (Lambda; asymmetry), M (Mu; median), and S (Sigma; variation coefficient). Estimated percentiles were p3, p5, p10, p15, p50, p85, p95, and p97. Statistical calculations were carried out on Excel worksheets and SPSS 16.0 software.

## Results

The anthropometric variables evaluated for the sample are presented in Table 1. The results show that below age 12, no significant differences occurred between both sexes in the variables studied (*p* < 0.05). However, differences emerged from age 13–18 (*p* < 0.05), for weight, stature, and WC. For BMI, these differences did not appear until age 17. Overall, females presented higher values for BMI in comparison to males during adolescence. On the other hand, males were taller and weighed more and had greater WC in relation to the females.

**Table 1:** Characterization of the sample.

**Age (Yr)**	***n***	**Weight (kg)**		**Height (cm)**		**BMI (kg/m**^****2****^**)**		**WC (cm)**	
		***X***	***SD***	***X***	***SD***	***X***	***SD***	***X***	***SD***
**MALE (*****n*** **= 4,473)**									
6.0–6.9	117	27.33	8.91	120.74	6.12	18.59	4.93	59.24	7.37
7.0–7.9	101	30.29	6.89	127.14	6.48	18.61	3.26	62.89	8.61
8.0–8.9	141	33.01	7.63	131.93	6.43	19.35	3.26	65.69	9.12
9.0–9.9	211	36.98	7.62	137.10	7.93	19.62	3.59	66.11	11.42
10.0–10.9	260	42.42	9.19	142.89	7.08	20.60	3.19	69.57	9.60
11.0–11.9	301	47.62	10.58	149.27	8.00	21.24	3.61	71.81	10.32
12.0–12.9	426	51.06	10.76	155.13	7.97	21.12	3.49	71.99	9.49
13.0–13.9	565	56.29	11.59	161.15	8.86	21.56	3.49	74.28	10.05
14.0–14.9	657	62.40	12.91	166.51	9.61	22.33	3.90	77.20	11.13
15.0–15.9	568	65.48	11.27	169.52	7.17	22.77	3.71	78.64	11.33
16.0–16.9	525	69.08	13.32	171.04	7.45	23.64	4.44	79.41	10.76
17.0–17.9	533	69.59	11.31	171.38	6.30	23.67	3.46	79.36	10.42
18.0–18.9	68	71.47	12.76	172.03	7.13	24.15	4.12	81.23	−9.20
Total	4473	56.83	16.62	159.23	15.87	21.94	4.01	74.57	11.62
**FEMALE (*****n*** **= 4,148)**									
6.0–6.9	121	27.07	6.63	120.64	8.39	18.44	2.93	59.05	7.07
7.0–7.9	147	30.52	8.13	127.48	8.40	18.50	2.88	61.91	8.11
8.0–8.9	155	31.84	7.74	130.23	6.47	18.86	3.33	64.33	8.87
9.0–9.9	223	38.74	9.13	139.29	7.51	19.78	3.55	66.68	10.93
10.0–10.9	312	42.89	9.54	144.46	7.22	20.42	3.47	68.47	9.99
11.0–11.9	295	47.23	8.96	149.87	7.14	20.94	3.25	70.63	8.62
12.0–12.9	395	52.05	11.63	154.89	5.82	22.07	4.56	72.68	9.01
13.0–13.9	397	54.86	9.88^*^	156.08^*^	7.08	22.52	3.73^*^	72.98	9.03^*^
14.0–14.9	568	58.37	10.72^*^	157.97^*^	6.87	23.35	3.88^*^	75.12	9.14^*^
15.0–15.9	434	59.65	11.30^*^	158.59^*^	5.02	23.67	4.07^*^	75.66	10.94^*^
16.0–16.9	512	60.99	12.21^*^	158.75^*^	6.75	24.29	6.23^*^	75.76	9.45^*^
17.0–17.9	517	60.63	11.65^*^	158.23^*^	7.26	24.36	6.95^*^	75.21	9.67^*^
18.0–18.9	72	60.96	9.74^*^	159.86^*^	6.01	24.46	3.60	75.27	7.20^*^
Total	4148	52.39	14.52^*^	151.88^*^	12.35	22.36	4.99^*^	71.97	10.49^*^

*Data are shown as mean (X) and standard deviation (SD)*.

*BMI, Body Mass Index; WC, Waist Circumference; Yr, Year*.

* *Significant differences in relation to male*.

### Physical Growth

**Figure 2:**
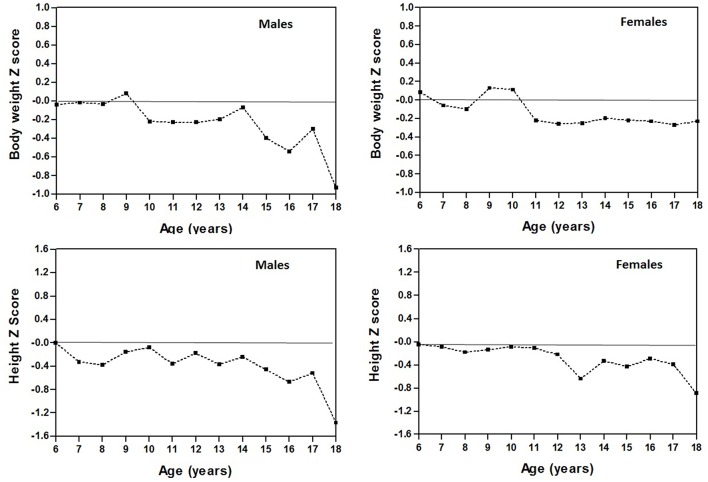
Comparisons of physical growth variables (weight and height) between students from the Maule Region (Chile) and the CDC-2012 references based on Z-scores.

Figure 2 shows comparisons of physical growth variables (weight and height) between students from the Maule Region (Chile) and the CDC-2012 references based on Z-scores. The results for weight represented a lower value than the CDC-2012 average with negative Z-score values ranging between −0.2 to −0.9 for males ages 10–18 and −0.22 to −0.27 for females 11–18 years old. In addition to values greater than the average between ages 9 and 10 years old in females with positive z-score values that varied between 0.10 and 0.13. Z-scores for height varied from −0.37 to −1.37 for males ages 13–18, and for females, Z-scores varied from −0.330 to −1.130 ages 12–18 years old. At earlier ages, the Z-score values were closer to zero. This represents a value closer to the average. In general, adolescents from the Maule Region showed less weight and height in comparison with the international reference.

**Figure 3:**
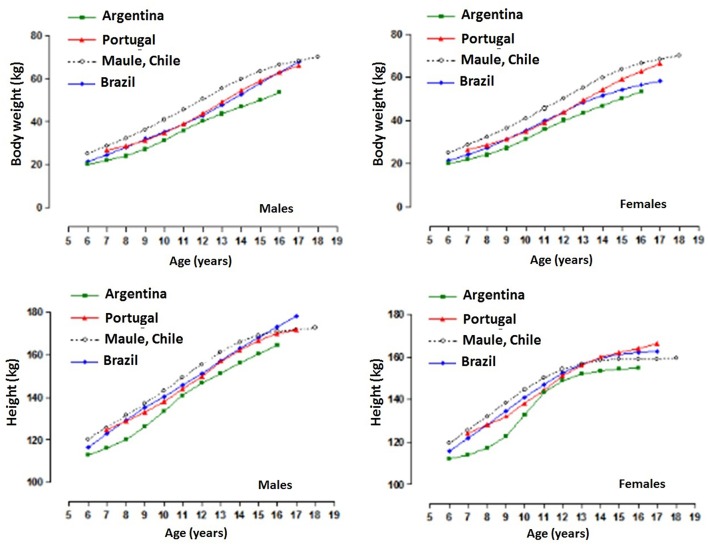
Comparison of physical growth variables (p50) of children and adolescents from the Maule Region (Chile) with international references.

The comparisons of physical growth variables (percentile 50) of the sample with the data from other international references are illustrated in Figure 3. Children and adolescents of both sexes had higher body weight in relation to the other references from ages 6–18. In terms of stature, males from the Maule Region (Chile) were taller from 6 to 14 years old, and until age 12 for females. In general, during adolescence, the sample studied showed stability in stature, yet slight discrepancies occurred in relation to other references.

### Body Adiposity

**Figure 4:**
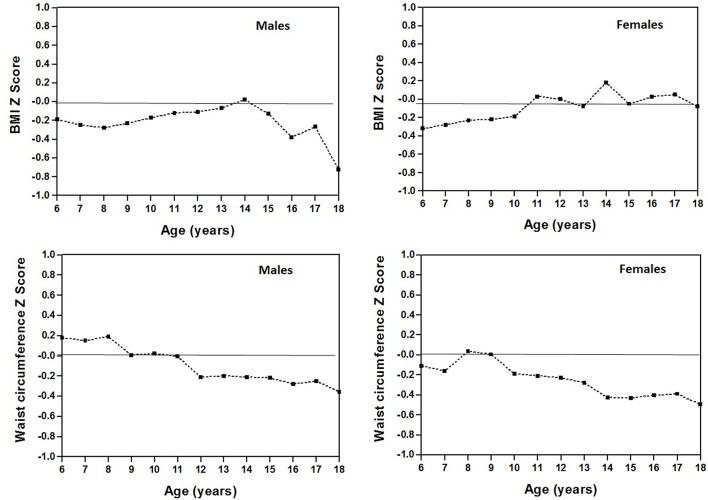
Comparisons of body adiposity variables (BMI and WC) between students from the Maule Region (Chile) and the CDC-2012 references based on Z-scores.

The comparisons of body adiposity (BMI and WC) between the CDC-2012 references and the sample are depicted in Figure 4 with the Z-scores. The Z-score values represent a lower BMI for the students studied in relation to the CDC-20‘2. The Z-score varied between −0.12 and −0.19 in males from 6 to 11 years old while for females, Z-scores varied from −0.17 to −0.32 ages 6–10 years old. In addition, females between the ages of 16 and 18 showed positive Z-score values ranging from 0.38 to 0.72. Both in the group of males 12–15 years old and in females ages 11–18, some similarities emerged with regard to the average values to the CDC-2012 reference with scores close to 0. With regard to WC, the Z-score values represented lesser value than the average of the CDC-2012 with negative z-score values that varied from −0.21 to −0.36 for males between the ages of 12–18 and −0.12 to −0.496 for females ages 10–18. At earlier ages (from 6 to 11 years old in males and from 6 to 9 years old in females), the Z-score values were close to 0. This represented a value equal to the average.

**Figure 5:**
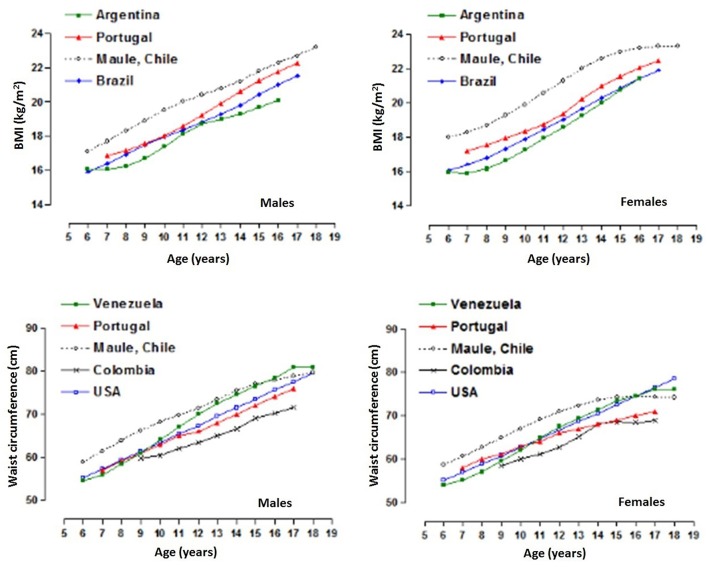
Comparison of body adiposity variables (P50) of children and adolescents from the Maule Region (Chile) with international references.

Comparisons of the BMI and WC between the sample and the international references are shown in Figure 5. The BMI percentile values (p50) of the Maule Region sample are higher in relation to the international references. The smallest difference occurred during adolescence. However, when compared with WC, the sample studied presented higher percentile values in relation to the other references. These comparisons were depicted graphically to illustrate the discrepancies between the 50th percentile values for each study.

Regional reference curves for growth (height and weight) and body adiposity (BMI and WC) for children and adolescents from ages 6.0–18.9 of both sexes are presented in Tables 2, 3. In both cases, the percentiles traditionally used for international references are highlighted (p3, p5, p10, p15, p50, p85, p90, p95, and p97).

**Table 2:** Percentile values for variables of physical growth (weight and height) of children and adolescents of both genders in the Maule Region (Chile).

**Age (Yr)**	**L**	**S**	**p3**	**p5**	**p10**	**p15**	**p50**	**p85**	**p90**	**p95**	**p97**	**L**	**S**	**p3**	**p5**	**p10**	**p15**	**p50**	**p85**	**p90**	**p95**	**p97**
	**Male (*****n*** **= 4,473)**											**Female (*****n*** **= 4,148)**										
**WEIGHT (kg)**																						
6.0–6.9	−1.00	0.22	17.8	18.5	19.7	20.5	25.3	32.8	35.3	39.8	43.4	−0.69	0.24	17.0	17.8	19.1	20.1	25.2	33.1	35.6	39.9	43.1
7.0–7.9	−0.70	0.22	19.9	20.8	22.2	23.2	28.7	36.9	39.5	43.8	47.1	−0.55	0.23	19.2	20.1	21.6	22.7	28.5	36.9	39.4	43.7	47.0
8.0–8.9	−0.43	0.22	22.1	23.1	24.8	26.0	32.4	41.2	43.8	48.1	51.3	−0.43	0.23	21.8	22.8	24.5	25.7	32.1	41.1	43.8	48.2	51.3
9.0–9.9	−0.21	0.22	24.5	25.7	27.7	29.2	36.4	46.0	48.7	53.1	56.2	−0.33	0.22	24.9	26.0	28.0	29.4	36.6	46.3	49.0	53.6	56.8
10.0–10.9	−0.05	0.22	27.3	28.7	31.0	32.7	40.9	51.3	54.2	58.7	61.9	−0.26	0.21	28.4	29.7	31.9	33.5	41.4	51.8	54.7	59.5	62.8
11.0–11.9	0.04	0.22	30.4	32.0	34.6	36.5	45.6	56.9	60.0	64.8	68.1	−0.22	0.20	32.0	33.5	35.9	37.6	46.2	57.3	60.3	65.3	68.7
12.0–12.9	0.08	0.21	33.8	35.5	38.4	40.5	50.4	62.6	65.8	70.9	74.4	−0.21	0.20	35.4	37.0	39.5	41.4	50.5	62.1	65.3	70.5	74.0
13.0–13.9	0.03	0.20	37.6	39.5	42.6	44.8	55.3	68.2	71.7	77.1	80.9	−0.24	0.19	38.3	39.9	42.6	44.5	53.9	65.9	69.2	74.5	78.2
14.0–14.9	−0.11	0.19	41.9	43.7	46.8	49.1	59.9	73.4	77.1	82.9	87.0	−0.33	0.18	40.6	42.2	44.9	46.8	56.3	68.6	72.1	77.5	81.4
15.0–15.9	−0.33	0.18	45.9	47.7	50.7	52.9	63.6	77.4	81.3	87.4	91.7	−0.51	0.18	42.4	43.9	46.5	48.4	57.8	70.3	73.9	79.6	83.8
16.0–16.9	−0.60	0.17	49.4	51.1	54.0	56.0	66.4	80.1	84.0	90.4	95.0	−0.75	0.18	43.6	45.1	47.6	49.4	58.5	71.1	74.8	80.9	85.3
17.0–17.9	−0.89	0.16	52.4	54.0	56.7	58.6	68.3	81.7	85.6	92.0	96.8	−0.99	0.17	44.5	45.9	48.2	49.9	58.7	71.3	75.0	81.4	86.2
18.0–18.9	−1.18	0.15	55.2	56.7	59.1	60.9	70.1	82.9	86.7	93.1	97.9	−1.24	0.17	45.2	46.5	48.7	50.4	58.8	71.2	75.1	81.8	86.9
**HEIGHT (m)**																						
6.0–6.9	−2.37	0.05	110.3	111.4	113.2	114.5	120.4	127.5	129.4	132.5	134.5	−4.51	0.06	108.9	110.0	111.7	113.0	119.3	128.3	131.1	135.9	139.6
7.0–7.9	−1.45	0.05	114.9	116.1	118.1	119.5	125.9	133.3	135.1	138.1	140.0	−2.93	0.06	114.5	115.6	117.6	119.0	125.6	134.0	136.4	140.2	142.9
8.0–8.9	−0.58	0.05	119.4	120.8	123.0	124.6	131.5	139.0	140.8	143.7	145.6	−1.36	0.05	120.0	121.4	123.5	125.0	132.0	139.9	142.0	145.1	147.3
9.0–9.9	0.15	0.05	124.1	125.7	128.1	129.8	137.1	144.8	146.7	149.5	151.3	0.13	0.05	125.7	127.2	129.6	131.3	138.5	146.0	147.9	150.6	152.4
10.0–10.9	0.70	0.05	129.2	130.9	133.6	135.4	143.1	151.0	152.8	155.6	157.4	1.49	0.05	131.1	132.9	135.5	137.3	144.6	151.8	153.5	156.0	157.5
11.0–11.9	1.23	0.05	134.7	136.6	139.4	141.4	149.4	157.3	159.2	161.9	163.7	2.64	0.05	136.1	137.9	140.8	142.6	150.0	156.8	158.4	160.6	162.1
12.0–12.9	1.87	0.05	140.3	142.3	145.4	147.4	155.6	163.6	165.4	168.0	169.8	3.50	0.04	140.1	142.1	144.9	146.8	154.1	160.6	162.1	164.2	165.5
13.0–13.9	2.56	0.05	145.7	147.8	151.0	153.1	161.4	169.2	170.9	173.5	175.1	4.01	0.04	143.1	145.0	147.9	149.7	156.8	163.0	164.4	166.4	167.6
14.0–14.9	3.14	0.05	150.4	152.6	155.8	157.9	166.1	173.6	175.2	177.6	179.2	4.06	0.04	145.1	147.0	149.7	151.5	158.3	164.3	165.6	167.5	168.7
15.0–15.9	3.54	0.04	154.0	156.1	159.2	161.3	169.2	176.3	177.9	180.2	181.6	3.76	0.04	146.5	148.2	150.8	152.4	158.9	164.7	166.0	167.9	169.1
16.0–16.9	3.72	0.04	156.4	158.5	161.5	163.4	170.9	177.7	179.2	181.3	182.7	3.30	0.04	147.3	148.9	151.3	152.8	159.0	164.7	166.0	167.9	169.1
17.0–17.9	3.72	0.04	158.3	160.1	162.9	164.7	171.9	178.3	179.7	181.7	183.0	2.82	0.04	147.8	149.3	151.6	153.1	159.0	164.6	165.9	167.8	169.0
18.0–18.9	3.66	0.04	159.9	161.7	164.3	165.9	172.6	178.7	180.0	182.0	183.2	2.35	0.03	148.4	149.8	151.9	153.4	159.2	164.7	166.0	167.8	169.0

*Yr, year; p, percentile*.

**Table 3:** Percentile values for body adiposity variables (BMI and WC) of children and adolescents in the Maule Region (Chile).

**Age (Yr)**	**L**	**S**	**p3**	**p5**	**p10**	**p15**	**p50**	**p85**	**p90**	**p95**	**p97**	**L**	**S**	**p3**	**p5**	**p10**	**p15**	**p50**	**p85**	**p90**	**p95**	**p97**
	**Male (*****n*** **= 4,473)**											**Female (*****n*** **= 4,148)**										
**BMI (kg/m**^**2**^**)**																						
6.0–6.9	−1.52	0.17	13.2	13.5	14.2	14.6	17.1	20.9	22.2	24.5	26.4	−0.30	0.16	13.4	13.9	14.7	15.3	18.0	21.3	22.3	23.7	24.7
7.0–7.9	−1.20	0.17	13.5	13.9	14.6	15.1	17.7	21.5	22.7	24.8	26.4	−0.38	0.16	13.7	14.2	15.0	15.5	18.3	21.7	22.7	24.2	25.3
8.0–8.9	−0.91	0.17	13.8	14.3	15.0	15.5	18.3	22.2	23.3	25.2	26.7	−0.47	0.16	14.0	14.5	15.3	15.9	18.7	22.3	23.3	24.9	26.0
9.0–9.9	−0.66	0.17	14.2	14.6	15.4	16.0	18.9	22.8	23.9	25.7	27.1	−0.57	0.16	14.5	15.0	15.8	16.4	19.3	23.0	24.0	25.7	26.9
10.0–10.9	−0.47	0.17	14.5	15.0	15.9	16.5	19.5	23.4	24.5	26.3	27.6	−0.65	0.16	15.1	15.6	16.4	17.0	19.9	23.8	24.9	26.7	28.0
11.0–11.9	−0.37	0.17	14.8	15.3	16.2	16.9	20.0	24.0	25.1	26.8	28.1	−0.72	0.16	15.7	16.2	17.0	17.6	20.6	24.7	25.8	27.7	29.1
12.0–12.9	−0.35	0.17	15.1	15.7	16.6	17.2	20.4	24.4	25.5	27.3	28.5	−0.82	0.16	16.3	16.8	17.7	18.3	21.3	25.5	26.7	28.7	30.2
13.0–13.9	−0.44	0.17	15.5	16.0	16.9	17.6	20.8	24.9	26.0	27.8	29.1	−0.95	0.16	16.9	17.4	18.3	18.9	22.0	26.4	27.6	29.8	31.3
14.0–14.9	−0.61	0.16	16.0	16.6	17.4	18.1	21.2	25.4	26.6	28.5	29.9	−1.13	0.16	17.5	18.0	18.9	19.5	22.6	27.1	28.4	30.7	32.4
15.0–15.9	0.85	0.16	16.7	17.2	18.0	18.6	21.8	26.0	27.2	29.3	30.8	−1.35	0.16	18.0	18.5	19.3	19.9	23.0	27.6	29.0	31.4	33.3
16.0–16.9	−1.13	0.15	17.3	17.8	18.6	19.2	22.3	26.5	27.8	30.0	31.6	−1.57	0.15	18.3	18.8	19.6	20.2	23.2	27.9	29.3	32.0	34.0
17.0–17.9	−1.43	0.15	18.0	18.5	19.2	19.8	22.7	27.0	28.3	30.6	32.3	−1.77	0.15	18.5	19.0	19.8	20.3	23.3	28.0	29.5	32.3	34.5
18.0–18.9	−1.73	0.14	18.7	19.1	19.8	20.4	23.2	27.4	28.7	31.1	32.9	−1.97	0.15	18.7	19.1	19.8	20.4	23.3	28.0	29.5	32.4	34.8
**WC (cm)**																						
6.0–6.9	−1.80	0.12	48.7	49.7	51.4	52.6	58.8	67.6	70.2	74.8	78.2	−1.77	0.12	48.8	49.8	51.4	52.6	58.7	67.1	69.7	74.0	77.2
7.0–7.9	−1.65	0.12	50.6	51.7	53.5	54.8	61.4	70.7	73.5	78.2	81.7	−1.68	0.12	50.3	51.3	53.0	54.3	60.6	69.5	72.1	76.5	79.9
8.0–8.9	−1.51	0.12	52.4	53.6	55.5	56.9	63.9	73.7	76.6	81.4	85.0	−1.59	0.12	51.8	52.9	54.7	56.1	62.7	71.9	74.6	79.2	82.6
9.0–9.9	−1.41	0.13	54.1	55.3	57.3	58.8	66.2	76,4	79.3	84.3	88.0	−1.51	0.12	53.5	54.6	56.5	57.9	64.9	74.4	77.2	81.8	85.3
10.0–10.9	−1.34	0.13	55.6	56.8	59.0	60.5	68.2	78.6	81.7	86.8	90.5	−1.43	0.12	55.2	56.4	58.4	59.8	67.0	76.8	79.6	84.3	87.8
11.0–11.9	−1.33	0.13	56.9	58.2	60.4	61.9	69.8	80.5	83.7	88.9	92.7	−1.35	0.12	56.8	58.1	60.1	61.6	69.1	79.0	81.9	86.7	90.2
12.0–12.9	−1.39	0.12	58.4	59.7	61.9	63.5	71.4	82.3	85.5	90.8	94.7	−1.28	0.12	58.3	59.6	61.7	63.3	70.9	81.1	84.0	88.8	92.3
13.0–13.9	−1.49	0.12	60.3	61.6	63.8	65.4	73.4	84.4	87.7	93.2	97.2	−1.24	0.12	59.5	60.8	63.0	64.6	72.4	82.7	85.7	90.5	94.0
14.0–14.9	−1.65	0.12	62.4	63.7	65.9	67.5	75.5	86.6	89.9	95.5	99.6	−1.24	0.12	60.5	61.9	64.1	65.7	73.6	84.1	87.0	91.9	95.4
15.0–15.9	−1.85	0.11	64.3	65.6	67.7	69.2	77.0	88.0	91.3	96.9	101.1	−1.27	0.12	61.2	62.5	64.7	66.4	74.3	84.8	87.7	92.7	96.2
16.0–16.9	−2.10	0.11	65.8	67.0	69.0	70.5	77.9	88.5	91.8	97.3	101.5	−1.35	0.12	61.6	62.9	65.1	66.7	74.5	84.9	87.9	92.8	96.4
17.0–17.9	−2.37	0.10	67.4	68.5	70.4	71.8	78.8	88.8	91.9	97.2	101.3	−1.47	0.11	61.8	63.1	65.2	66.8	74.4	84.7	87.6	92.5	96.1
18.0–18.9	−2.65	0.09	69.0	70.0	71.8	73.1	79.7	89.1	92.0	97.0	100.8	−1.58	0.11	62.0	63.2	65.3	66.8	74.2	84.3	87.2	92.1	95.7

*BMI, Body Mass Index; WC, Waist Circumference; Yr, year; p, percentile*.

## Discussion

This current study is the first one in Chile that examined physical growth and body adiposity variables of students, both children and adolescents, in the Maule Region (Chile). Regarding physical growth, our results showed that students in the Maule Region presented a similar growth pattern during childhood. However, during adolescence, students had lower weight and height values when compared with their counterparts in the CDC-2012 international reference (11). This means that the children and adolescents studied reached the CDC-2012 reference points in childhood, but in adolescence, the growth standards were still far apart even though the Chilean population has presented important demographic, social, and economic changes during the last decades (19). Effectively, the social and health indicators, as well as the proportion of families below the poverty line, infant mortality, and life expectancy have improved notably in recent years (20).

On the other hand, when compared graphically with other international studies, the students examined here showed greater weight and even greater height from the early teens. Later, height values became relatively similar to regional curves in Argentina, Brazil, and Portugal.

Generally, several international studies with similar goals compared data with the CDC-2000 (3, 16, 21, 22) and the CDC-2012 (12) references. These studies corroborate our findings. Our research results have shown differences in physical growth patterns of children and adolescents. These findings may be related to intrinsic and extrinsic factors because in addition to genetic factors, socio-economic, and cultural differences between countries influence physical growth.

In fact, these features directly reflect the nutritional, environmental, and biological maturation (23) factors. It is also possible that specific aspects of the surroundings, both constructed and natural, combined with nutritional trends, attention to public health, and physical activity levels. These may have influenced the differences that occurred (24).

With regard to the comparisons of body adiposity with the CDC-2012 references, the results indicate that the students studied showed differences in both BMI and WC. The average values of the study did not surpass those of the reference, especially during childhood for BMI, and for WC during adolescence.

These discrepancies found lead to the assumption that the students studied are approaching the reference values. These indicate a clear path toward similarity to the CDC-2012 standards. On the other hand, when the values of BMI and WC were compared with international studies (3, 5, 12, 16), we observed that students of both sexes showed higher values of BMI in all age groups, WC included. This pattern is visible until age 12 in males and age 14 in females in such a way that the discrepancies with other studies were relatively minor during adolescence.

These findings may result from the differences in genetic constitution (3) and lifestyle among populations. For example, those findings related to sedentary lifestyle, irregular food intake, and greater fat consumption (25, 26). Generally, these are the factors that lead to drastically change the patterns of body adiposity. In addition, these factors often present themselves in societies experiencing the phenomenon of nutritional transition that is specific to developing countries. This is the specific case of Chile where the parameters of adiposity are increasing due to the adoption of the modern lifestyle (27).

Consequently, based on the results obtained from this research, the discrepancies between the CDC-2012 (11) reference and the international research (3, 5, 12, 16) led us to develop curves to identify the weight, height, BMI, and WC of children and adolescents in the Maule Region (Chile). In fact, a number of these previous studies highlighted differences and discrepancies between international percentiles (3, 16, 28, 29). These studies confirm the need to propose regional curves.

Overall, physical growth and body adiposity percentiles provide relevant information for inferring the nutritional and health status of children and adolescents (30). As a result, the LMS method was used to generate percentiles (18). Currently, this methodology is considered to be refined, powerful, and robust for calculating and estimating the inter-individual variability. In addition, this method has shown clear advantages over other estimation methods (31).

The cut-off points adopted in this study for growth and body adiposity were based on international references (7, 9, 11). The reference data generated in our study may be useful for diagnosing and classifying children and adolescents by adopting the following categories according to age and sex: (*p* < 10, p10 to p85, p85 to p95, and *p* > 95). Although the definition of overweight in growing populations is somewhat arbitrary (32), as far as is known, no consensus about the indicators or parameters used to define obesity exists, let alone the cut-off points to use. Furthermore, currently, all of the existing reference standards, whether national or international, are subject to systematic errors (16).

In this sense, the regional percentiles proposed in our study were not created to be definitive for values of weight, height, BMI, and WC for each child and/or adolescent, but rather to represent the typical growth and adiposity levels that the regional sample of Maule presented at the time of this research. Moreover, the proposed reference values may be used to compare other regional samples of Chile with the results from this present study. The actual values of the curves, generally, help to determine to what degree the physiological needs are being met during the growth process and motor development (33).

Considering the lack of data on growth and body adiposity in Chile, the regional references that we proposed may be of significant assistance for pediatricians, nutritionists, and professionals involved in the growth and development of children and adolescents. The focus of this study may be analyzed from the clinical and epidemiological perspective, and even from the researcher's point of view to compare and/or contrast with other samples and/or contexts. In addition, this study is relevant since the results obtained may help identify the physical growth and body adiposity patterns with less bias than the CDC-2012 international reference curves.

One strength of this study was the large database used for the sample. Also, it was collected in a probabilistic manner in such a way that the generalizability of the results to similar contexts becomes possible. In addition, the precision and accuracy criteria were taken into account during and before the anthropometric evaluations were conducted (34). Another strength of this research was the use of the Z-score method to compare the physical growth and body adiposity variables with the CDC references. Use of Z-score values is considered to be more precise for evaluating individuals and populations (35). Consequently, it is preferred, and its use is suggested in research contexts for monitoring the nutritional status of children and adolescents (35, 36).

This study also has some limitations. One is the absence of controlling for biological maturation, especially sexual maturation. In Chile, its use and application is limited due to socio-cultural reasons. On the other hand, the proposed percentiles will require periodic updating in order to maintain the height rhythm and ethnical variation within the population studied. This would only be possible if future studies were longitudinal.

To conclude, in this study, we found discrepancies with the CDC-2012 curves and the international studies, both for physical growth variables and body adiposity. In addition, percentiles are proposed to estimate weight, height, BMI, and WC for children and adolescents of the Maule Region (Chile). This information may be the basis for elaborating and implementing the new references at a national level. Calculations may be made quickly and accurately by using the following link: http://www.reidebihu.net/body_adiposity_ch.php. Finally, the results from this study may be useful for pediatricians, nutritionists, physical education teachers, and researchers in general.

## Data Availability

The datasets generated for this study are available on request to the corresponding author.

## Ethics Statement

This study was approvals by the Ethics Committee from the Universidad Autónoma of Chile (Protocol No. 238/2014) and received the authorization of the respective authority of the educational centers.

## Author Contributions

MC-B and RG-C conceived and designed the study. JP-C and CL-R performed the experiments. MA and DL-P contributed reagents, materials, and analysis tools. RG-C and MC-B performed the statistical analysis. MC-B, RG-C, MA, and DL-P drafted the manuscript. CA participated in the drafting, translation, and correction of the manuscript. All authors edited and revised the manuscript with critical feedback given.

### Conflict of Interest Statement

The authors declare that the research was conducted in the absence of any commercial or financial relationships that could be construed as a potential conflict of interest.
